# A Case Report of Short‐Term Metastasis After Surgery for Mammary Paget's Disease

**DOI:** 10.1002/ccr3.71585

**Published:** 2025-12-07

**Authors:** Chao Wang, Baiyi Yan, Jing Wang, Jiahui Zhou, Dongxu Li, Xiaofang Gao, Yong Pan

**Affiliations:** ^1^ Department of Breast Cancer Beijing Arion Cancer Center Beijing China; ^2^ Department of Lung Cancer Beijing Arion Cancer Center Beijing China

**Keywords:** ductal carcinoma in situ, HER2‐positive, Mammary Paget's disease, metastasis, toxicity management

## Abstract

Mammary Paget's disease (MPD) with ductal carcinoma in situ (DCIS) pathology can metastasize rapidly. HER2‐positive status likely drives aggression, warranting early postoperative systemic imaging and close surveillance. Anti‐HER2 agents require proactive toxicity management. Personalized dose adjustments and multidisciplinary management are essential to optimize outcomes.

## 1 Introduction

Mammary Paget's disease (MPD) is a rare presentation of breast malignancy, accounting for approximately 1%–3% of all primary breast cancers [1]. It is characterized by eczematous changes of the nipple‐areolar complex (NAC), often associated with itching, erythema, and ulceration. Sir James Paget first described the condition in 1874, noting the ominous association between this chronic nipple eruption and underlying breast carcinoma [2].

The histopathological hallmark of MPD is the presence of large, pale‐staining Paget cells within the epidermal layer of the nipple. The pathogenesis is widely explained by the epidermotropic theory, which proposes that these cells are ductal carcinoma cells that migrate from an underlying malignancy in the breast parenchyma through the lactiferous ducts to the nipple epidermis [3, 4]. This theory is strongly supported by the fact that over 90% of MPD cases are associated with an underlying ductal carcinoma in situ (DCIS) or invasive carcinoma [5, 6].

Clinically, MPD can be classified into three categories: Paget's disease with invasive ductal carcinoma (PD‐IDC), Paget's disease with DCIS (PD‐DCIS), and—in very rare instances—Paget's disease of the nipple alone [7]. Accordingly, the prognosis of MPD is intrinsically linked to the nature of the underlying tumor. The National Comprehensive Cancer Network (NCCN) guidelines classify PD‐DCIS as a noninvasive breast cancer, similar to DCIS alone, which typically carries an excellent prognosis with a 5‐year disease‐free survival exceeding 95% [8, 9]. The standard management often involves surgery, with options ranging from nipple‐areola complex resection to mastectomy, with or without radiotherapy [10, 11, 12]. For PD‐DCIS, the role of adjuvant systemic therapy is limited, and the risk of systemic recurrence is considered negligible.

However, the clinical course of MPD is not always straightforward. Underlying HER2‐positive disease, which is highly prevalent in MPD, has been implicated in more aggressive behavior [13, 14]. While rapid systemic recurrence is a recognized feature of PD‐IDC, it is an exceptionally rare and devastating event in patients with a pathological diagnosis of pure PD‐DCIS.

Herein, we report a highly instructive and paradoxical case of a patient who presented with classic MPD and was postoperatively diagnosed with HER2‐positive DCIS without histological evidence of invasion. Contrary to all expectations for a noninvasive cancer, she developed widespread systemic metastases within 18 months of surgery. This case challenges the conventional diagnostic and therapeutic paradigm for PD‐DCIS and compellingly highlights the potential for the HER2‐positive MPD‐DCIS phenotype to demonstrate virulent, metastatic potential. We document the successful management of this aggressive recurrence with anti‐HER2 therapy, alongside the navigation of life‐threatening treatment‐related toxicities, providing critical insights for clinical practice.

## 2 Case History and Examination

A 51‐year‐old woman presented in January 2019 with eczematous changes of the right NAC, clinically diagnosed as MPD. She subsequently underwent a right breast skin‐sparing mastectomy with immediate reconstruction at an external institution. Postoperative pathological examination of the mastectomy specimen confirmed the presence of DCIS, histological grade II, with no evidence of invasive carcinoma. Sentinel lymph node biopsy was negative (0/4). Immunohistochemistry (IHC) revealed an ER‐negative, PR‐negative, HER2‐positive (3+) phenotype with a high Ki‐67 index of 80%. The patient received no adjuvant systemic therapy post‐surgery.

In July 2020, follow‐up at the same institution discovered that the right axillary lymph node was enlarged. Later, pain gradually appeared in the right femur, left scapula, and sternum, for which no further workup was performed at that time. In May 2021, the patient experienced persistent pain in the right femur, left subscapularis, and sternum. A follow‐up PET‐CT scan revealed multiple bone metastases throughout the body, ground glass shadows in both lungs suggesting cancerous lymphangitis, enlarged lymph nodes in the right axilla suggesting metastasis, and liver nodules not excluding metastasis.

For further treatment, the patient was transferred to our hospital in June 2021. She had a history of bilateral amazingel injection, hysteromyoma surgery, denies family history of breast and ovarian cancer, father died of bladder cancer. Physical examination showed stable vital signs, wheelchairing, postoperative changes in the right breast, no obvious lumps in both breasts, palpable enlarged lymph nodes in the right axilla and groin, fixed, without tenderness, no dry or wet rales in both lungs, heart rate 96 beats/min, arrhythmia, soft abdomen, liver, spleen subcostal not reached, and lower limbs not swollen. Based on these findings, the patient was preliminarily diagnosed as postoperative Paget's disease of the right breast with secondary malignancies in the right axillary lymph node, bone, lungs, and liver.

**FIGURE 1:**
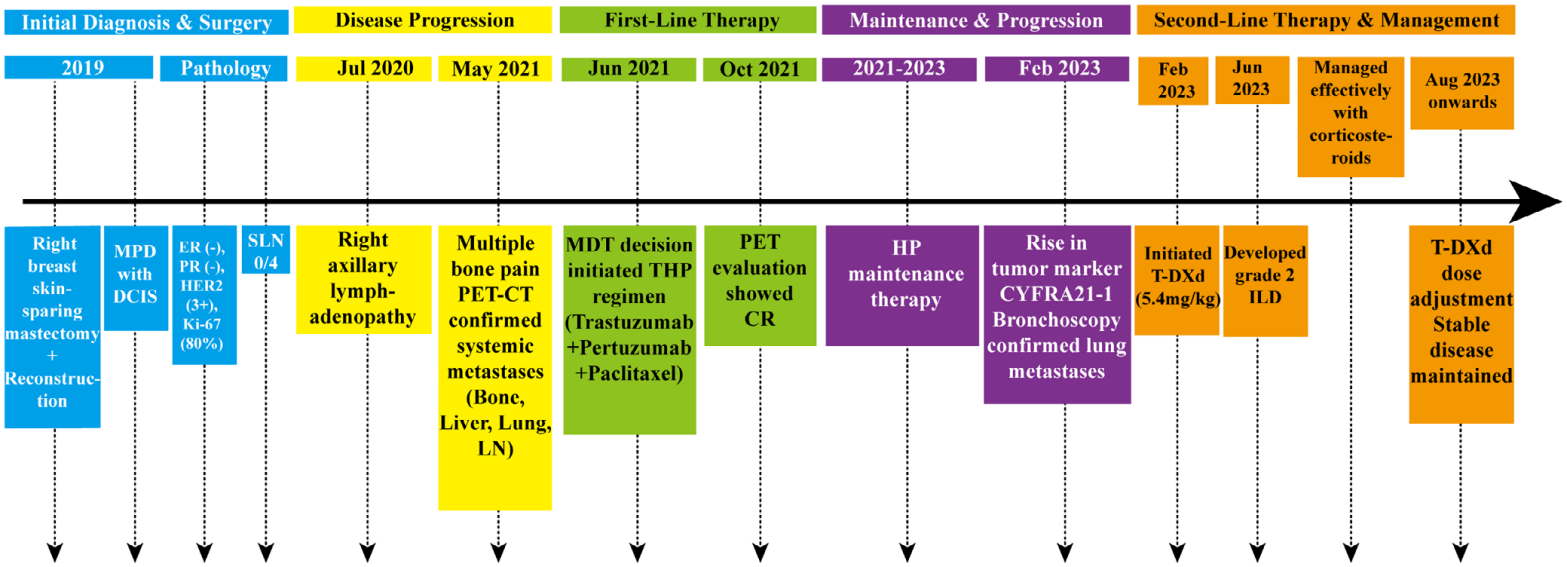
Timeline.

The chronological sequence of the patient's clinical course is summarized in Figure 1, which provides a visual overview of key events from initial diagnosis to management of metastatic disease.

## 3 Differential Diagnosis, Investigations, and Treatment

**FIGURE 2:**
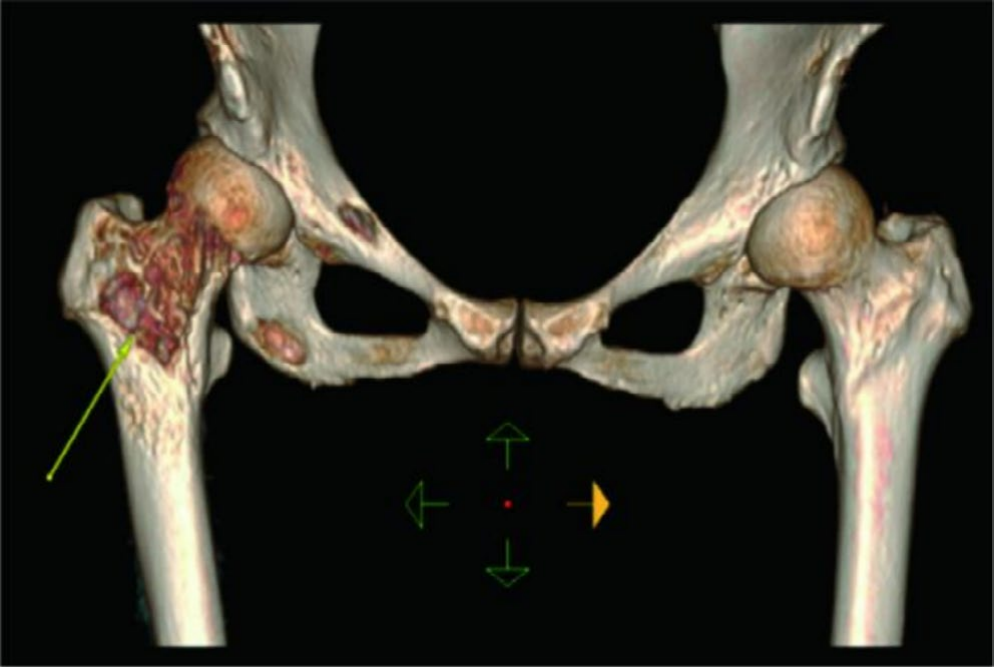
CT reconstruction of right femur metastasis.

The patient underwent comprehensive abdominal ultrasound and systemic superficial lymph node ultrasound, which suggested possible liver metastasis. Multiple enlarged lymph nodes were found in the right neck area 4 and 5, subclavian, and right inguinal region, suggesting metastasis. The primary diagnostic challenge was to determine whether the multiple metastases originated from an occult breast cancer or a second primary tumor. On 6 June 2021, a multidisciplinary meeting (MDT) was convened. The consensus was that the multiple bone metastases, particularly in the right femur which posed a high risk of pathological fracture (Figure 2), represented the most immediate threat. However, a definitive histological diagnosis was prioritized.

**FIGURE 3:**
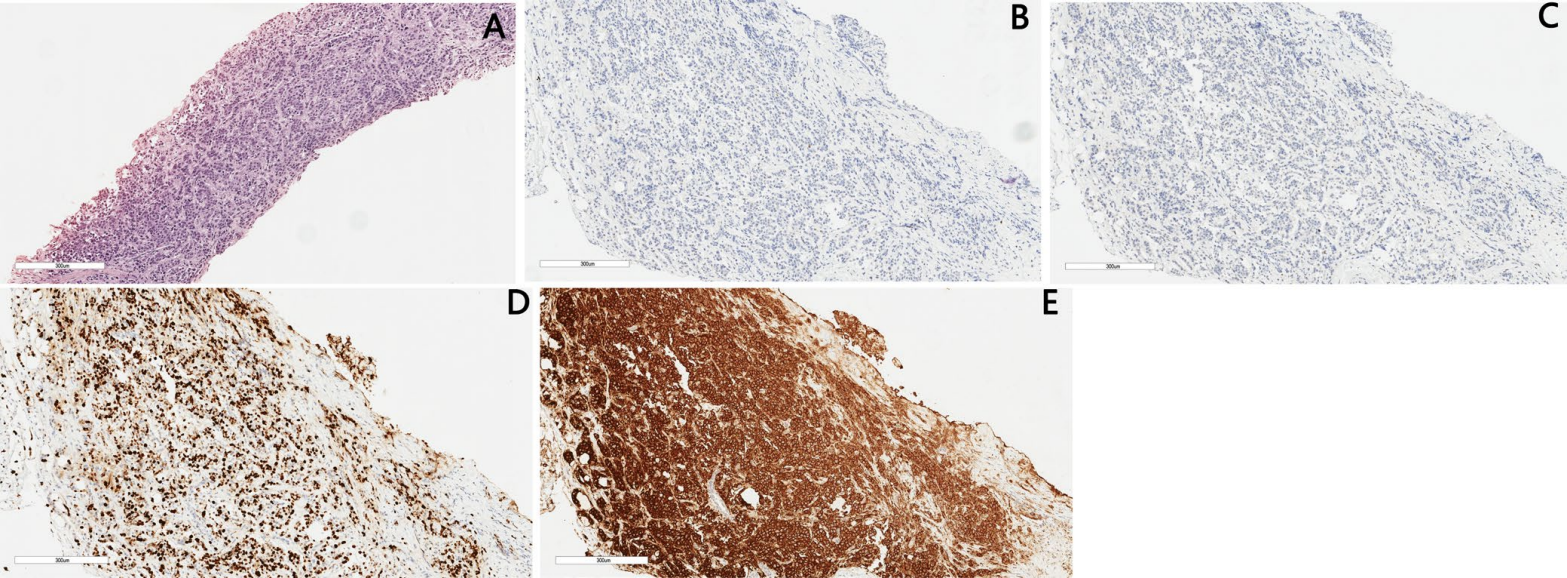
Right inguinal lymph node biopsy (Scale bar, 300 μM). (A) Microscopic picture (H&E stain); (B) Negative estrogen receptor (ER) immunostaining; (C) Negative progesterone receptor (PR) immunostaining; (D) Ki‐67 labeling index, 40%; (E) Her2‐positive cells immunostaining.

An ultrasonography‐guided biopsy of the right inguinal lymph node was performed on 7 June 2021 (the right axillary lymph node was not biopsied due to extensive necrosis). The pathology confirmed metastatic carcinoma consistent with a breast origin. Immunohistochemistry (IHC) revealed an ER‐negative, PR‐negative, HER2‐positive (3+) phenotype with a Ki‐67 index of approximately 40% (Figure 3). Although the histological sections from the initial 2019 surgery were unavailable for review, the IHC profile of the metastatic lesion was identical to the primary DCIS, confirming the origin. The final diagnosis was adjusted to: Recurrent Paget's disease of the right breast → cTxN3M1, HER2‐positive invasive breast cancer, with metastases to bone, liver, lungs, and lymph nodes.

The subsequent MDT on June 11th debated the treatment sequence: orthopedic surgery for the femoral lesion versus immediate systemic therapy. It was decided that while femoral surgery was necessary, systemic anti‐HER2 dual‐target therapy (trastuzumab + pertuzumab) should be initiated during the preoperative period.

**FIGURE 4:**
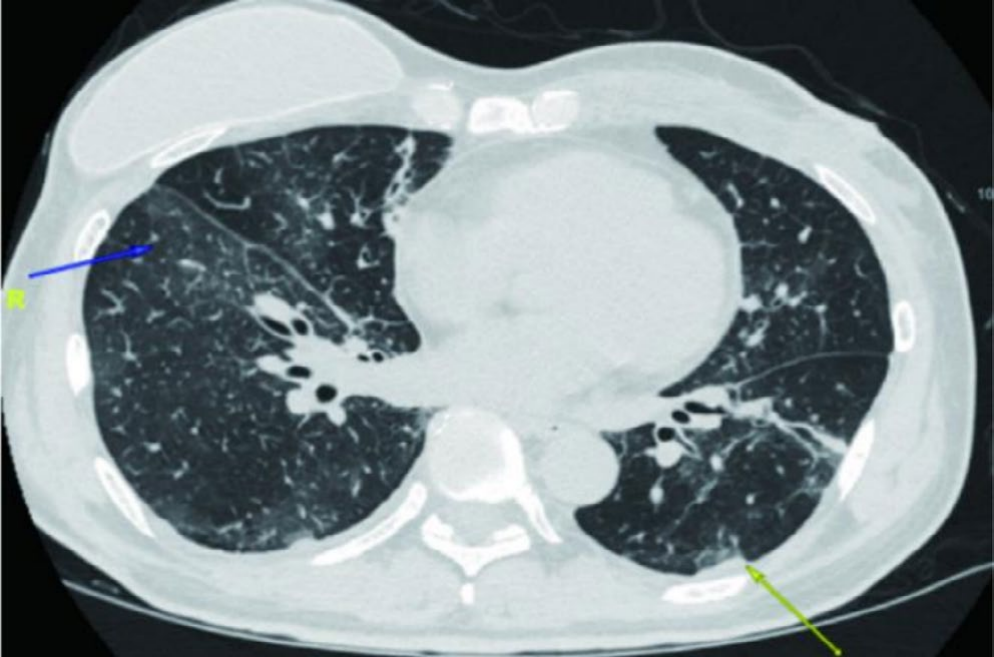
Bilateral interstitial inflammation with subpleural exudation and thickening on chest CT.

The first cycle of dual‐target therapy was administered on June 11th, 2021. Approximately 10 min after initiating the trastuzumab infusion (following pertuzumab), the patient developed cough, chills, high fever, vomiting, and diarrhea. Despite treatment with 5 mg IV dexamethasone, cough and chest tightness persisted. A CT pulmonary angiogram excluded pulmonary embolism, but a chest CT revealed new interstitial inflammation in both lungs (Figure 4).

An emergency MDT on June 13 concluded that the interstitial pneumonia was likely related to the drug infusion rather than tumor progression or infection. Given that trastuzumab is a cornerstone of HER2‐positive breast cancer treatment, desensitization was recommended after the pulmonary symptoms improved without glucocorticoid treatment.

**FIGURE 5:**
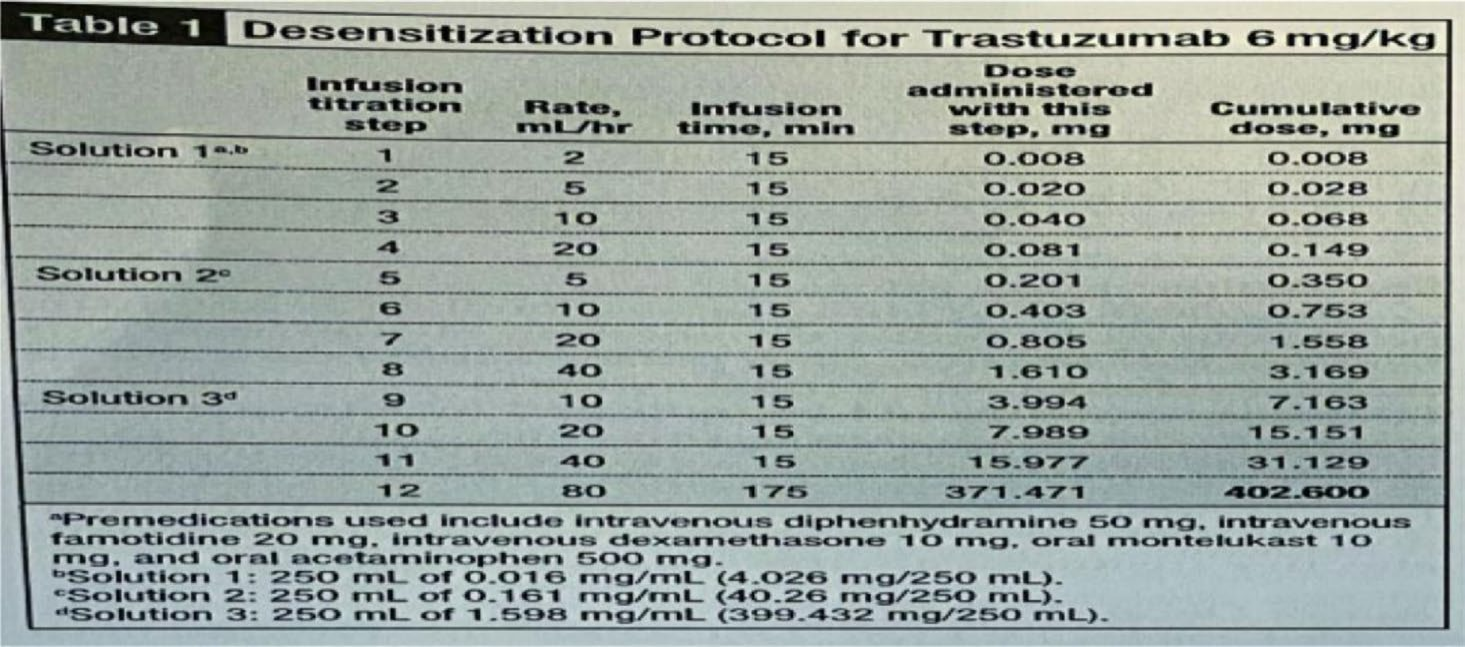
Desensitization treatment with trastuzumab.

On June 17, 2021, desensitization to the THP regimen (paclitaxel liposome, trastuzumab, pertuzumab) was successfully initiated in the ICU (Figure 5). The process was well‐tolerated, and the patient subsequently transitioned to the standard dose. Treatment‐related toxicities included Grade I digestive tract reactions, Grade I constipation, Grade II bone marrow suppression, and Grade II alopecia. Cardiac ejection fraction (EF) remained stable between 63% and 65%. Concurrently, denosumab 120 mg subcutaneously every 4 weeks was initiated for bone metastasis.

**FIGURE 6:**
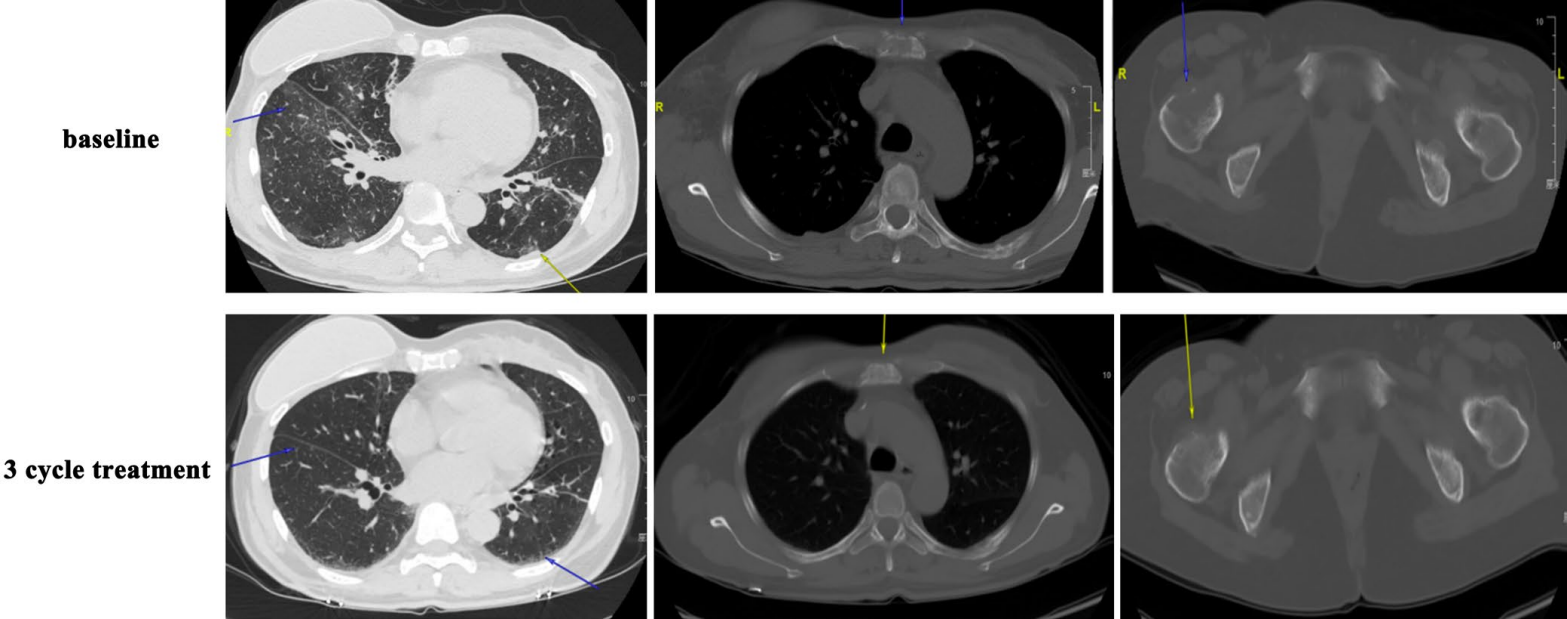
CT evaluation after first‐line THP treatment.

**FIGURE 7:**
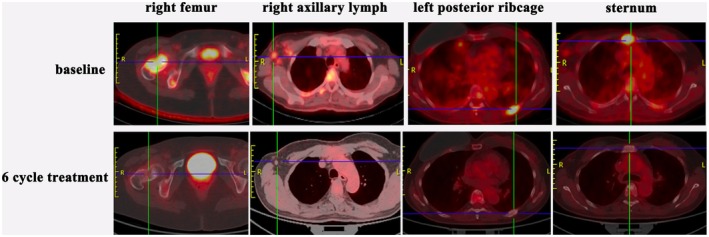
PET‐CT evaluation after first‐line THP treatment.

**FIGURE 8:**
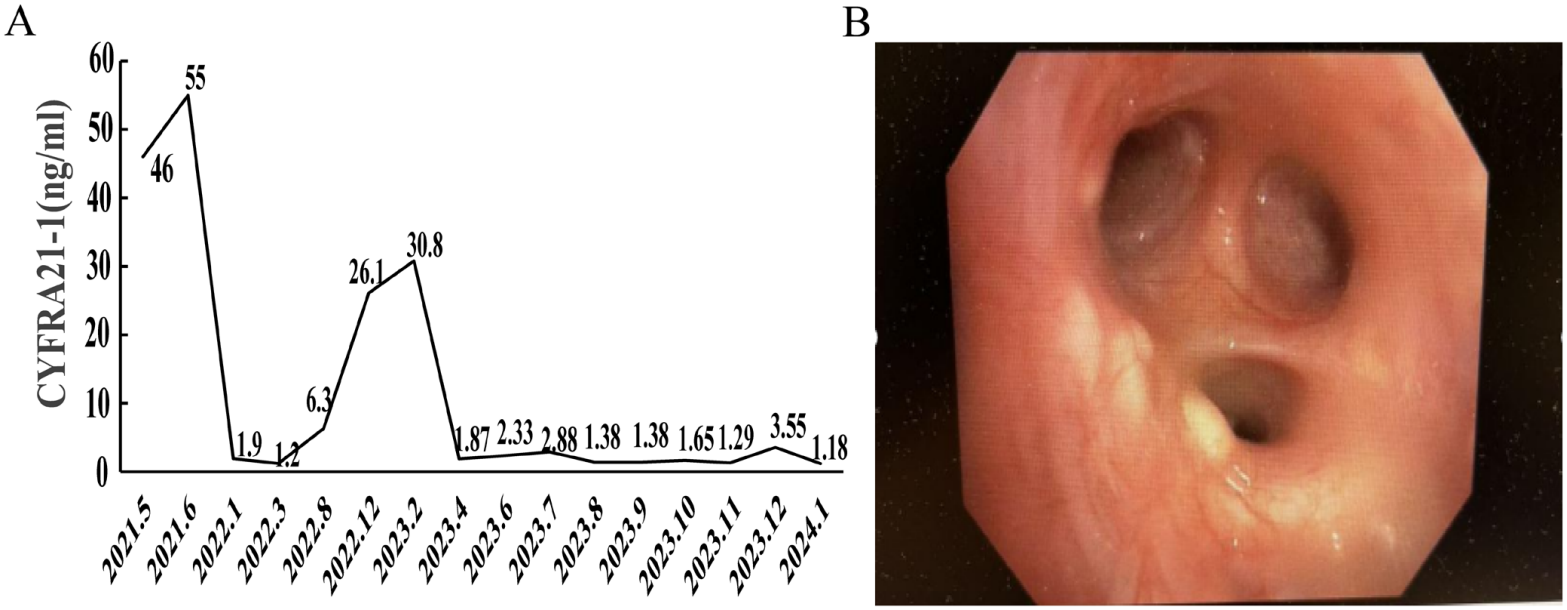
(A) Trend of CYFRA21‐1 index. (B) Bronchoscopy‐metastasis of left lung.

The patient responded remarkably to first‐line therapy. A CT evaluation in July 2021 showed a partial response (PR, Figure 6), and a PET‐CT in October 2021 confirmed a complete response (CR, Figure 7). After 7 cycles of THP, the regimen was de‐escalated in November 2021 to HP (trastuzumab + pertuzumab) maintenance therapy due to cumulative chemotherapy‐related toxicity. During this period, the serum tumor marker CYFRA21‐1 was identified as a sensitive indicator of disease status (Figure 8A).

**FIGURE 9:**
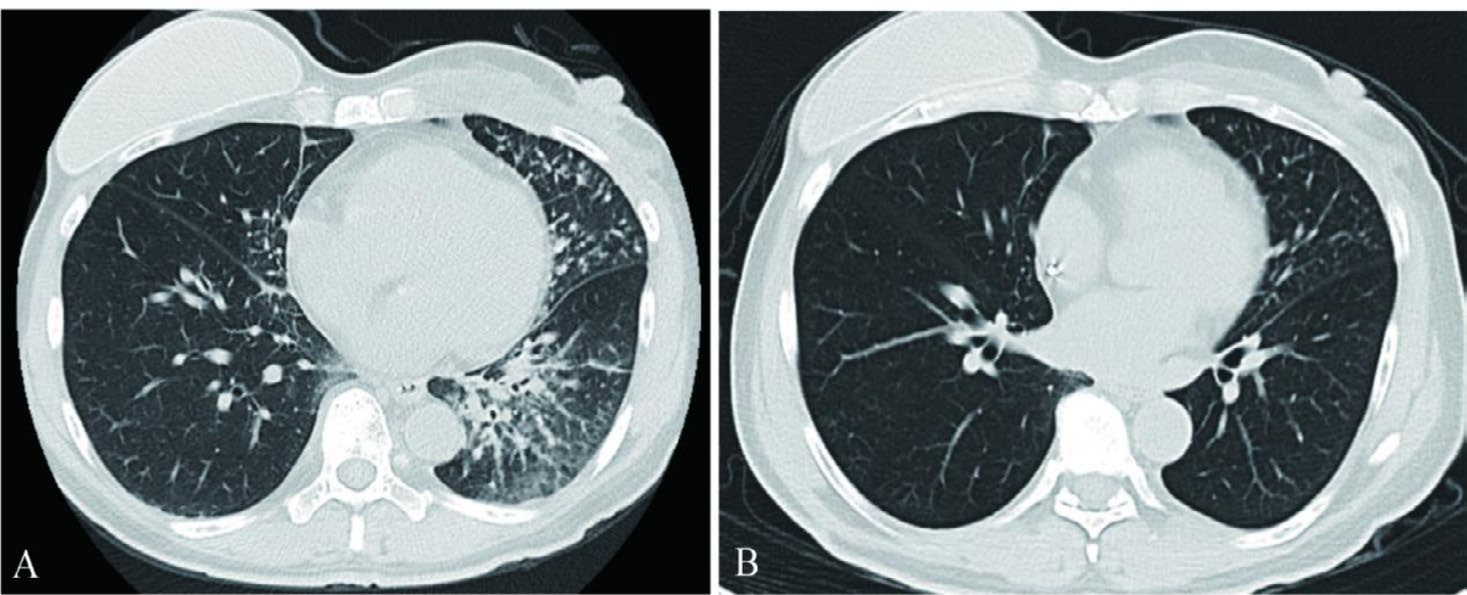
(A) Chest HRCT (recurrent CT findings in the left lung–interstitial pneumonia). (B) Chest HRCT (after 3 cycles of T‐DXd treatment).

The patient remained in remission for 18 months. In February 2023, a significant rise in CYFRA21‐1 was observed. A bronchoscopy revealed a miliary nodule (Figure 8B), and a chest HRCT showed interstitial lung disease (ILD) changes (Figure 9A). Pathology from a bronchoscopic biopsy confirmed HER2‐positive (3+) metastatic breast cancer. Based on this evidence of disease progression, second‐line therapy with Trastuzumab Deruxtecan (T‐DXd) was initiated on February 14, 2023, at a dose of 5.4 mg/kg.

**FIGURE 10:**
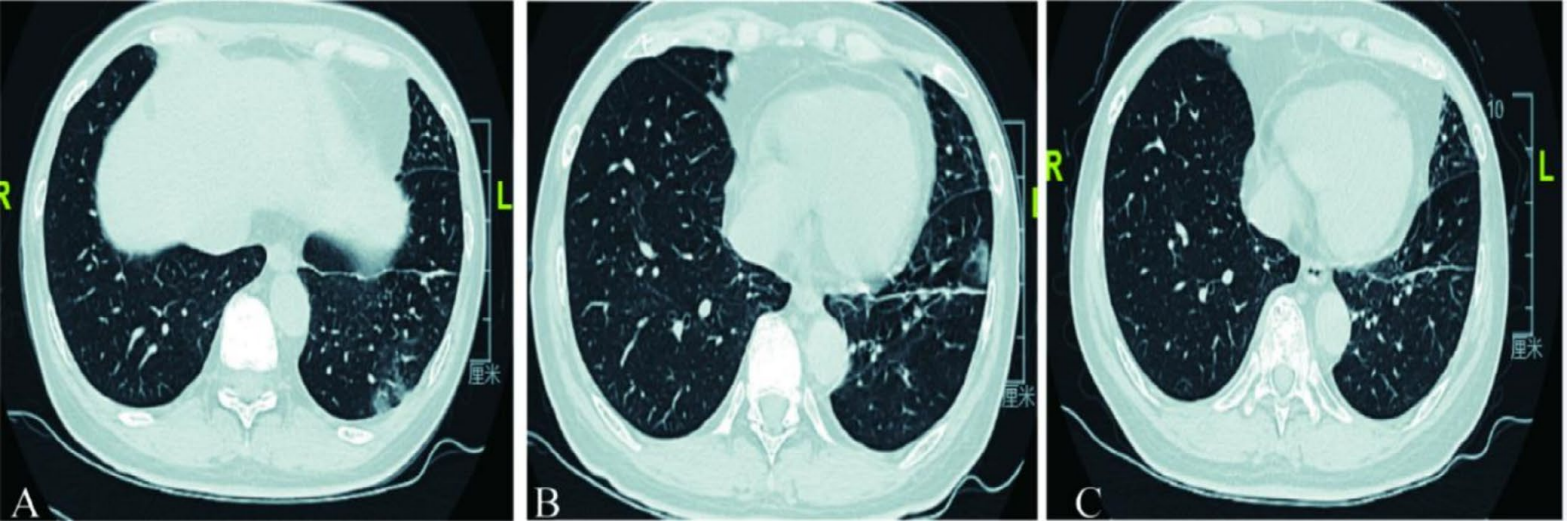
(A, B) HRCT (2nd degree ILD). (C) HRCT (ILD remission after methylprednisolone treatment).

The patient experienced Grade 2 nausea and vomiting during the first cycle, prompting a dose reduction to 4.4 mg/kg from the second cycle onwards, alongside a quadruple antiemetic regimen, which improved tolerance. A CT scan after 3 cycles confirmed a partial response (PR, Figure 9B). However, after the 6th cycle (May 2023), the patient developed a cough and shortness of breath. A HRCT in June 2023 revealed Grade 2 ILD (Figure 10A,B). Oral prednisone was started but provided insufficient relief, and the patient was hospitalized with hypoxemia (SO2 83%). She received intravenous methylprednisolone (40 mg twice daily for 3 days), followed by a tapering dose of oral prednisone, alongside prophylaxis for pneumocystis jirovecii pneumonia. The interstitial inflammation gradually improved, with near‐complete resolution by August 2023 (Figure 10C).

Following another MDT discussion, T‐DXd was cautiously reintroduced on August 30, 2023, at a further reduced dose of 3.2 mg/kg, with low‐dose prednisone maintenance. Due to steroid intolerance (fatigue, poor sleep), the prednisone was discontinued in September 2023, and the T‐DXd dose was reduced to 1.6 mg/kg for cycles 8–11. In December 2023, with stable disease and no ILD recurrence, the T‐DXd dose was adjusted to 2.7 mg/kg every 4 weeks for better tolerability. The patient tolerated this regimen well, with only Grade 1–2 digestive tract reactions managed by a four‐drug antiemetic regimen including olanzapine.

## 4 Conclusion and Results

A 51‐year‐old woman with HER2+ metastatic breast cancer achieved a complete response (CR) after first‐line THP (trastuzumab/pertuzumab/paclitaxel), maintaining 18 months of progression‐free survival. Upon recurrence, T‐DXd induced a partial response but caused ILD, successfully managed with steroids and dose reduction. The key findings from this case demonstrate that dual HER2‐targeted therapy provides durable disease control in metastatic breast cancer, while T‐DXd shows efficacy but requires dose modifications for ILD management, with CYFRA21‐1 proving valuable for disease monitoring and proactive toxicity management enabling continued therapy.

## 5 Discussion

The rapid and widespread metastatic recurrence in our patient, initially diagnosed with HER2‐positive MPD associated with pure DCIS, presents a profound clinical paradox. This case not only challenges the conventional biological paradigm that metastatic potential is exclusive to invasive carcinoma but also provides critical insights into the aggressive nature of the HER2‐positive MPD‐DCIS phenotype. Furthermore, the successful navigation of life‐threatening toxicities from anti‐HER2 therapy underscores the importance of proactive and multidisciplinary management in achieving long‐term survival.

The pathogenesis of MPD offers a crucial framework for understanding its aggressive potential. The dominant “Epidermotropic Theory” posits that Paget cells originate from an underlying ductal carcinoma and migrate to the nipple epidermis, supported by the high frequency of concomitant DCIS or invasive cancer [3, 5]. This theory inherently assigns MPD cells a migratory and invasive phenotype from the outset. Central to this biology is the HER2 oncogene. Large‐scale studies confirm HER2 positivity as a hallmark of MPD, with rates of 59%–78% reported, the highest among breast cancer subtypes [4, 15, 16]. A xenograft model has demonstrated that HER2‐positive breast cancer cells can specifically colonize the nipple epidermis, recapitulating MPD [17]. Therefore, in our patient, the HER2‐positive (3+) status is likely the fundamental driver of both the initial Paget's lesion and its devastating systemic progression.

While the aforementioned evidence explains the aggressiveness of HER2‐positive MPD in general, our case presents a more radical challenge. The patient's initial pathology rigorously confirmed DCIS only, with no microinvasion identified. This aggressive potential, though rare, is corroborated by other case reports. Kar et al., in their single‐center series, reported that while none of their patients with pure DCIS died, one patient with MPD‐DCIS did develop ipsilateral axillary lymph node metastasis 3 years postoperatively [18]. More strikingly, Sueta et al. described a case with a clinical course remarkably similar to ours: a patient with HER2‐positive MPD with DCIS and dermal microinvasion experienced systemic recurrence (including carcinomatous lymphangiosis of the lung) just 18 months after mastectomy, and also achieved a complete response with taxane‐trastuzumab combination therapy [19].

Our patient's course demonstrating—widespread systemic metastasis within a similar 18‐month timeframe, but without any documented microinvasion—extends these observations. As summarized in Table 1, these cases collectively illustrate a spectrum of metastatic behavior in HER2‐positive MPD‐DCIS. Our case, positioned at the most aggressive end of this spectrum, suggests that the HER2‐positive MPD‐DCIS phenotype itself can possess metastatic potential, and that its virulence can equal or even surpass that of cases with documented microinvasion.

**TABLE 1:** Reported cases of Mammary Paget's disease (MPD) with DCIS and aggressive or metastatic behavior.

Author (year)	Cases (*n*)	Underlying pathology	HER2 status	Recurrence/metastasis timing	Site of recurrence/metastasis	Documented microinvasion
Present case	1	DCIS	Positive (3+)	18 months	Systemic (bone, lung, liver, lymph nodes)	No
Sueta et al. (2010) [19]	1	DCIS with intraductal spread	Positive (3+)	18 months	Systemic (contralateral breast, chest wall, lung lymphangitis, lymph nodes)	Yes (dermal)
Kar et al. (2021) [18]	1	DCIS	Not specified for this case	3 years	Locoregional (axillary lymph node)	Not specified (implied none)

Emerging molecular evidence provides a potential mechanism for this observation. A recent study found an exceptionally high co‐expression rate of HER2 and the RNA‐binding protein MSI1 in MPD, with MSI1 expression positively correlating with lymph node metastasis [20]. This cooperation between HER2 and other molecular pathways may confer an invasive capability that is not apparent in conventional histology.

The successful outcome in this case was twofold: leveraging effective anti‐HER2 therapy and meticulously managing its severe toxicities. The severe infusion reaction to trastuzumab threatened to deprive the patient of this cornerstone therapy. Guided by evidence that most reactions are manageable non‐IgE‐mediated events [21, 22], a rapid desensitization protocol was successfully implemented, enabling treatment continuity and initial complete response.

Subsequently, the development of T‐DXd‐induced ILD presented a more formidable challenge. The successful management involved prompt drug interruption, aggressive corticosteroid therapy, and most importantly, personalized dose reduction. Rechallenging at 50% of the standard dose and further individualizing based on tolerance exemplifies a modern oncological approach that maintains efficacy while prioritizing safety [23].

In conclusion, this case compellingly argues that the HER2‐positive MPD‐DCIS phenotype itself can drive metastatic potential, potentially overriding the traditional distinction between DCIS and invasive disease. For patients with HER2‐positive MPD‐DCIS, the excellent overall prognosis of DCIS should not induce complacency. First, diagnostically, it should be regarded as a potentially systemic disease from inception, with pathologists encouraged to extensively sample specimens and clinicians interpreting HER2‐positive MPD‐DCIS diagnoses in this setting with extreme caution. Second, severe toxicities to essential anti‐HER2 agents, including infusion reactions and ILD, can often be managed through desensitization, aggressive supportive care, and personalized dose modifications, allowing patients to derive sustained benefit from these potent therapies. This case ultimately champions a paradigm of precision oncology that encompasses both precise biomarker‐driven treatment selection and precise, proactive management of the resulting toxicities.

## Author Contributions

**Chao Wang:** conceptualization, methodology, supervision. **Baiyi Yan:** data curation, formal analysis, investigation, methodology, writing – original draft. **Jing Wang:** conceptualization, investigation. **Jiahui Zhou:** investigation, methodology. **Dongxu Li:** investigation, methodology. **Xiaofang Gao:** conceptualization, methodology. **Yong Pan:** methodology, supervision, writing – review and editing.

## Funding

The authors have nothing to report.

## Ethics Statement

The case report has been submitted for Ethical Board Review and approved as an ethically sound report.

## Consent

Written informed consent was obtained from the patient for publication of this case report and any accompanying images. A copy of the written consent is available for review by the Editor‐in‐Chief of the journal.

## Conflicts of Interest

The authors declare no conflicts of interest.

## Data Availability

The data are available from the corresponding author on reasonable request.
